# The interaction between instrumental activities of daily living and dual sensory function on cognition among the elderly in China: A cross‐sectional survey

**DOI:** 10.1002/ibra.12124

**Published:** 2023-08-09

**Authors:** Heting Liang, Zhixia Jiang, Xiaoling Yang, Shuang Li, Xiaoling Zhao, Yongya Dai, Siqin Liu, Yumeng Zhang, Xiaoli Yuan

**Affiliations:** ^1^ Department of Nursing Affiliated Hospital of Zunyi Medical University Zunyi Guizhou China; ^2^ College Office Guizhou Nursing Vocational College Guiyang Guizhou China; ^3^ Faculty of Nursing Guizhou Nursing Vocational College Guiyang Guizhou China; ^4^ Faculty of Nursing Zunyi Medical University Zunyi Guizhou China

**Keywords:** cognition, elderly, hearing, instrumental activities of daily living, interaction, vision

## Abstract

To explore the interaction of instrumental activities of daily living (IADLs) and dual sensory function on cognition in the elderly. A cross‐sectional survey was conducted in six general hospitals in China, from October 2022 to December 2022. Data collection included general information, IADLs scale, self‐reported sensory function questionnaire, and mini‐mental state examination (MMSE). Binary logistic regression was used to examine the association between factors and cognition. The interactive effect was evaluated by synergy index (S), relative excess risk due to interaction (RERI), and attributable proportion due to interaction (AP). The odds ratio (OR) of IADLs decline in cognition is 4.412 (95% confidence interval [CI]: 3.633–5.358, *p* < 0.001); the OR of dual sensory difficulty on cognition is 2.502 (95% CI: 1.272–4.921, *p* = 0.008). The OR of interaction between IADLs decline and dual sensory difficulty on cognition is 13.737 (95% CI: 9.726–19.400, *p* < 0.001). RERI (95% CI) = 7.823 (3.230–12.417), AP (95% CI) = 0.570 (0.392–0.747), S (95% CI) = 2.593 (1.616–4.160). IADLs decline and dual sensory difficulty are associated with cognitive decline. IADLs decline and dual sensory difficulty have interaction with cognitive decline; the interaction is greater than the sum effect of those two on cognitive decline independently. Sensory and IADLs assessment can be used as early screening items for cognition among the elderly. In addition, protecting sensory function and maintaining IADLs in the elderly can help protect their cognition.

## Graphical abstract

The aim of this study was to explore the interaction between instrumental activities of daily living (IADL) and dual sensory function in the elderly and its correlation with cognition. Logistic regression was conducted, and the relative excess risk ratio, attribution ratio, and interaction index (S) were calculated to examine the effects of IADLs, sensory function, and their interaction on cognition among the elderly. Results indicated that there was no multiplicative interaction between IADL decline and dual sensory difficulty on the risk of cognitive decline in the elderly, but there was additive interaction.

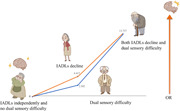

## 1 INTRODUCTION

As the elderly population grows, the health‐related problem of the elderly has become a research hotspot. Cognitive impairment is one of the major diseases that affected the daily life and health of the elderly.^1^, ^2^ The main causes of cognitive impairment of the elderly are changes in brain structure and function caused by aging, diseases, or side effects of drugs,^3^, ^4^, ^5^ which not only have a negative impact on the health and health‐related quality of life of the elderly but also increase the demand for care and burden for the public medical health resource.^6^, ^7^ However, there is no effective medical treatment for cognitive impairment;^8^ therefore, other interventions are needed to protect the cognitive function of seniors, which maintains their health and quality of life, and reduces the social and economic burden.

Previous studies^9^, ^10^, ^11^ have demonstrated that the ability of the elderly to perform activities of daily living is related to their cognitive function. However, compared to the basic activities of daily living (BADLs), instrumental activities of daily living (IADLs) are more complex abilities that individuals need to have to live independently, such as going shopping, cooking, washing clothes, and mode of transportation. IADLs are more sensitive to small functional defects than BADLs, so IADLs are usually impaired earlier than BADLs.^12^ Early screening of IADLs can help identify early declines in cognitive function in seniors.

Sensory function decline is also caused by aging, in which vision and hearing especially affect the daily life of the elderly. Studies^13^, ^14^, ^15^ have shown that visual and hearing difficulties in seniors can negatively influence their cognitive function. The elderly with visual difficulty would have problems with seeing objects, which further affects their perception and understanding of the surrounding environment.^16^ Similarly, hearing difficulty would make it difficult for the elderly to hear sounds and languages, which affects their auditory perception and understanding of the surrounding environment.^17^ In addition, visual and hearing difficulties of the elderly may affect their mental health, leading to problems such as loneliness and depression, which further affect their cognitive function. It has been examined that difficulty in dual sensory (vision and hearing) has greater negative effects on their cognitive function.^13^, ^14^, ^15^, ^17^

To our knowledge, there was no research that has included IADLs, dual sensory difficulty, and cognitive function into one model to explore the correlation and further interaction. On this basis, this study focused on the analysis of the relationship between IADLs, dual sensory, and cognition of the elderly, and further explored whether they have interactive effects on cognition. The results of this study support that the elderly at high risk of cognitive decline may be identified by sensory and IADLs assessment so that the cognition of high‐risk groups can be further evaluated and the awareness of active treatment of high‐risk groups can be improved.

## 2 METHODS

### 2.1 Sample

This study employed a cross‐sectional survey conducted in six general hospitals in Zunyi City, Guizhou Province, from October 2022 to December 2022, using a convenience sampling method. Inclusion criteria are as follows: (i) age ≥60 years old; (ii) be able to communicate; and (iii) informed consent and voluntary participation in the study. Exclusion criteria: (i) patients with severe mental illness; (ii) patients with severe and terminal diseases. According to the Kendall sample estimation method for multivariate analysis, the minimum sample size was required to be 10 times the number of variables.^18^ The questionnaire applied in the study included 40 variables, and the minimum sample size for this survey was therefore 400. This study has been approved by the Ethics Review Committee of the Affiliated Hospital of Zunyi Medical University on October 28, 2022 (KLL2022‐814).

### 2.2 Measurement and variables

#### 2.2.1 Quality control

The evaluators were trained and qualified before conducting an assessment. Before the assessment, the evaluators explained the purpose and summary content of this research to the participants. After obtaining the consent, evaluators asked them with unified guidance and filled in the questionnaire based on the participants' answers. All data were checked by two researchers; those illogical questionnaires were removed to ensure the validity of the questionnaires.

#### 2.2.2 Demographics and health profile

This includes gender, age, marital status, level of education, total number of chronic diseases, exercise habits, and self‐reported health.

#### 2.2.3 Instrumental activities of daily living

**Figure 1:**
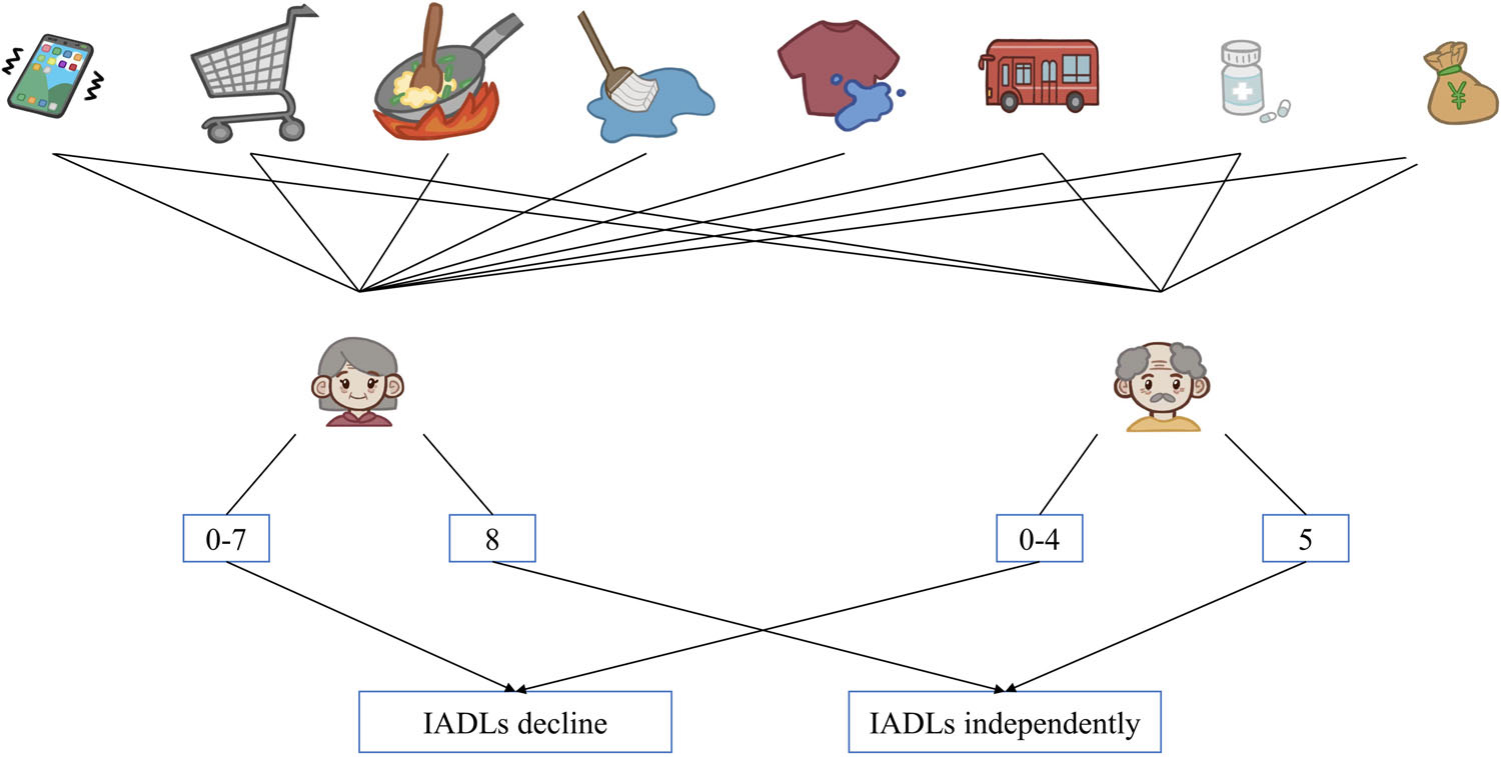
Assessment of instrumental activities of daily living (IADLs). [Color figure can be viewed at wileyonlinelibrary.com]

IADLs were measured by using the Lawton Instrumental Activities of Daily Living Assessment Scale,^19^ which includes eight items: the ability to use the telephone, shop, food preparation, housekeeping, laundry, mode of transportation, responsibility for own medications, ability to handle finances, with each activity being scored as one point for being able to complete independently and zero points for the opposite. To avoid potential gender differences, men were not assessed on food preparation, housekeeping, and laundry,^20^ so the range of scores was 0 to 5 for men and 0 to 8 for women. The full score indicates that the elderly had independent IADLs; otherwise, it indicates declines in IADLs among the elderly (Figure 1).

#### 2.2.4 Self‐reported vision and hearing function

**Figure 2:**
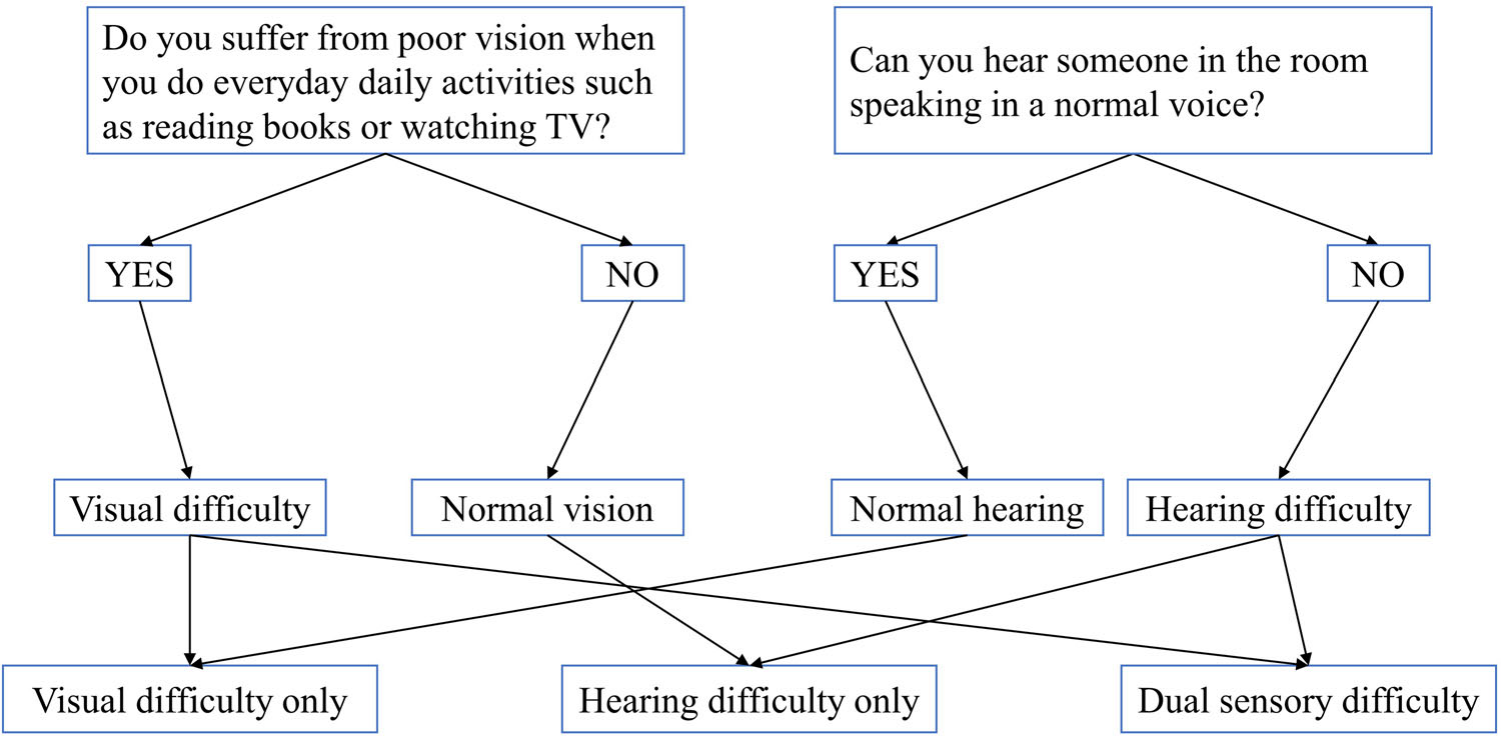
Assessment of sensory function. [Color figure can be viewed at wileyonlinelibrary.com]

This questionnaire includes two questions. Question 1: “Do you suffer from poor vision when you do daily activities such as reading books or watching TV?” The options were set to “yes” and “no,” participants who answer “yes” are considered to be visual difficulty. Question 2: “Can you hear someone in the room speaking in a normal voice?” The options were set to “yes” and “no,” participants who answered “no” were considered to have hearing difficulty. Those who self‐reported both visual and hearing difficulties were considered as having “dual sensory difficulty” (Figure 2).

#### 2.2.5 Cognition

The Mini‐Mental State Examination (MMSE)^21^ was adopted to measure the cognitive function of the elderly, a total of 30 items, including time and place orientation, immediate memory, attention, delayed memory, and verbal and written ability. One point for one correct answer. The score ranges from 0 to 30 points. Cognitive decline was defined by the following criteria: illiteracy ≤17 points, primary school ≤20 points, junior high school, and above ≤24 points.

### 2.3 Statistical analysis

Excel and SPSS 27.0 (IBM) were used for statistical analysis. Quantitative data were described by using mean and standard deviation, while qualitative data were described as frequencies and percentages (%). Besides, the *χ* ^2^ test was used to compare the difference between groups. Logistic regression was conducted to analyze the effects of IADLs, sensory function, and their interaction on cognition among the elderly, with the test level *α* = 0.05, expressed in odds ratios (ORs) and 95% confidence interval (CI). According to the evaluation method proposed by Rothman,^22^ the ability of IADLs, sensory function, and the product term of those two, were included in the logistic regression for the multiplicative interactions, and if the confidence interval (95% CI) of the product term contained 1, there was no multiplicative interaction; the Excel table prepared by Andersson et al.^23^ was applied to calculate the relative excess risk ratio (RERI), attribution ratio (AP), and interaction index (S) to exam the additive interaction, and if the 95% CI of RERI and AP contained 0 and the 95% CI of S contained 1, then there was no additive interaction.

## 3 RESULTS

### 3.1 Participants' characteristics

A total of 3853 seniors were included in the study. The mean age of participants was (71.44 ± 7.25) years old, ranging from 60 to 110 years old. Among the participants, 51.73% were male; 71.55% of the participants had primary school education or below, and most of the participants (86.37%) were married; more than half of the participants had at least one chronic disease (63.35%) and exercise habits (63.17%); most of the participants (92.94%) had no dual sensory difficulty. Compared with those with normal cognitive function, participants with cognitive decline were more likely to be female, more than 70 years and older, had primary school education or less, unmarried, had two or more chronic diseases, had no exercise habits, self‐reported poor health status, had IADLs decline, and had dual sensory difficulty (*p* < 0.001) (Table 1).

**Table 1:** Characteristics of respondents with normal cognition and with cognitive decline.

Variable	Total (%)	Cognitive decline (%)	*χ* ^2^	*p*
Gender			18.977	<0.001***
Male	1993 (51.73)	465 (23.33)		
Female	1860 (48.27)	549 (29.52)		
Age (years)			183.918	<0.001***
60–69	1733 (44.98)	301 (17.37)		
70–79	1533 (39.79)	449 (29.29)		
80 years old and above	587 (15.23)	264 (44.97)		
Level of education			43.958	<0.001***
Primary level and below	1977 (51.31)	550 (27.82)		
Junior level	780 (20.24)	213 (27.31)		
High school level and above	1096 (28.45)	251 (22.90)		
Marital status			15.430	<0.001***
Married	3328 (86.37)	839 (25.21)		
Unmarried	525 (13.63)	175 (33.33)		
Total number of chronic diseases			28.192	<0.001***
None	1412 (36.65)	318 (22.52)		
One	1450 (37.63)	377 (26.00)		
Two or more	991 (25.72)	319 (32.19)		
Exercise habits			52.534	<0.001***
Yes	2434 (63.17)	545 (22.39)		
No	1419 (36.83)	469 (33.05)		
Self‐reported health status			140.918	<0.001***
Good	1024 (26.58)	172 (16.80)		
General	2413 (62.62)	646 (26.77)		
Poor	416 (10.80)	196 (47.12)		
IADLs decline			467.190	<0.001***
No	1816 (47.13)	183 (10.08)		
Yes	2037 (52.87)	831 (40.80)		
Only visual difficulty			0.095	0.758
No	2851 (73.99)	754 (26.45)		
Yes	1002 (26.01)	260 (25.95)		
Only hearing difficulty			2.235	0.135
No	3405 (88.37)	883 (25.93)		
Yes	448 (11.63)	131 (29.24)		
Dual sensory difficulty			178.023	<0.001***
No	3581 (92.94)	849 (23.71)		
Yes	272 (7.06)	165 (60.66)		

Abbreviation: IADLs, instrumental activities of daily living.

*** *p* < 0.001.

### 3.2 The IADLs, dual sensory and cognition of participants

Among the 3853 seniors who participated in our study, IADLs decline was found in 2037 (52.87%) participants, 272 (7.06%) reported that they had dual sensory difficulty, and 1014 (26.32%) had cognitive decline (Table 2).

**Table 2:** Cognitive decline in different exposure groups. (%) of participants.

IADLs decline	Dual sensory difficulty	Total (*n* = 3853)	Normal cognition (*n* = 2839)	Cognitive decline (*n* = 1014)
No	No	1765 (45.81%)	1594 (90.31%)	171 (9.69%)
Yes	No	1816 (47.13%)	1138 (62.67%)	678 (37.33%)
No	Yes	51 (1.32%)	39 (76.47%)	12 (23.53%)
Yes	Yes	221 (5.74%)	68 (30.77%)	153 (69.23%)

*Note*: No IADLs decline means IADLs independently. No dual sensory difficulty means no difficulty with vision and/or hearing.

Abbreviation: IADLs, instrumental activities of daily living.

### 3.3 Association between IADLs, dual sensory function, and cognition among the elderly

The results of logistic regression analysis showed that after controlling confounding factors (gender, age, education background, marital status, total number of chronic diseases, exercise habits, self‐reported health status), IADLs decline (OR = 4.63, 95% CI: 3.828–5.612) and self‐reported dual sensory difficulty (OR = 3.013, 95% CI: 2.273–3.994) are associated with cognitive decline (*p* < 0.001), as shown in Table 3.

**Table 3:** Association between IADLs, dual sensory function, and cognition.

Variable	*B*	*p*	OR (95% CI)
Female (Ref. = male)	0.298	<0.001***	1.347 (1.145–1.585)
Age (Ref. = 60–69 years)			
70–79 years old	0.285	0.002*	1.329 (1.107–1.596)
80 years and above	0.734	<0.001***	2.083 (1.649–2.631)
Education background (Ref. = primary level and below)			
Junior level	0.609	<0.001***	1.838 (1.476–2.289)
High school level and above	‐0.350	0.029*	0.705 (0.514–0.966)
Unmarried (Ref. = married)	−0.103	0.377	0.902 (0.718–1.134)
Total number of diseases (Ref. = none)			
One	−0.032	0.740	0.968 (0.800–1.171)
Two or more	0.088	0.416	1.092 (0.884–1.348)
No exercise habits (Ref. = exercise)	0.176	0.039*	1.192 (1.009–1.409)
Self‐reported health status (Ref. = good)			
General	0.227	0.031*	1.255 (1.021–1.541)
Poor	0.653	<0.001***	1.922 (1.433–2.579)
IADLs decline (Ref. = IADLs independent)	1.534	<0.001***	4.635 (3.828–5.612)
Dual sensory difficulty (Ref. = no dual sensory difficulty)	1.103	<0.001***	3.013 (2.273–3.994)

Abbreviations: CI, confidence interval; IADLs, instrumental activities of daily living; Ref., reference.

* *p* < 0.05

*** *p* < 0.001.

### 3.4 Interaction of IADLs and dual sensory on cognition

#### 3.4.1 Multiplicative interaction of IADLs and dual sensory on cognition

By further including the product terms of IADLs and self‐reported dual sensory function as independent variables in the above logistic regression model, the result showed the multiplicative interaction was not significant (*p* = 0.557), as shown in Table 4.

**Table 4:** Multiplicative interaction of IADLs and dual sensory on cognition.

Variable	*B*	*p*	OR (95% CI)
IADLs decline (Ref. = IADLs independent)	1.519	<0.001***	4.569 (3.753–5.562)
Dual sensory difficulty (Ref. = normal)	0.917	0.009*	2.503 (1.263–4.961)
IADLs decline × dual sensory difficulty	0.225	0.557	1.252 (0.592–2.65)

Abbreviations: CI, confidence interval; IADLs, instrumental activities of daily living.

* *p* < 0.05

*** *p* < 0.001.

#### 3.4.2 Additive interaction of IADLs and dual sensory on cognition

**Figure 3:**
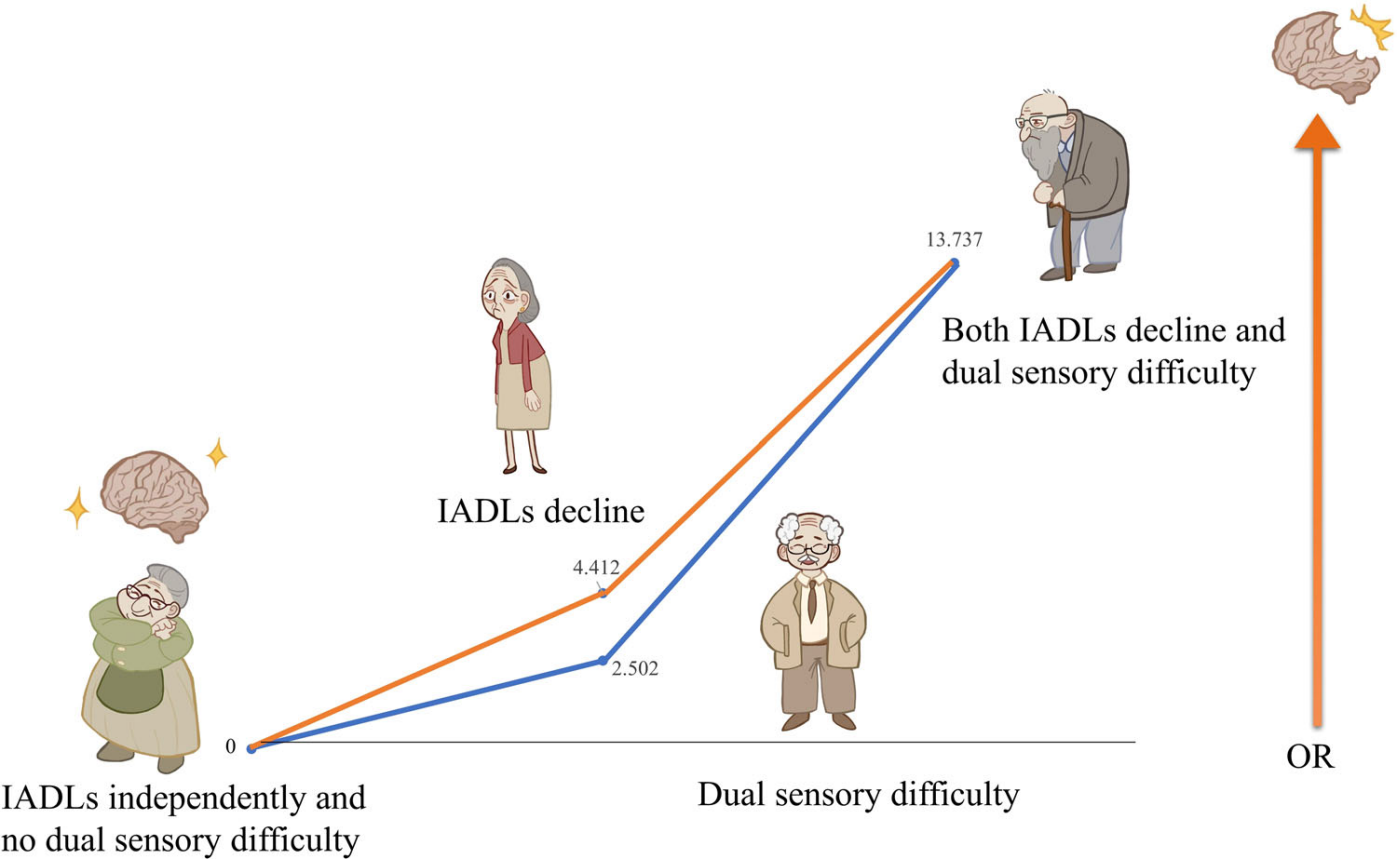
The odds ratio (OR) of cognitive decline with contributions of different exposure categories marked (IADLs decline and dual sensory difficulty). The reference category is IADLs independently and no dual sensory difficulty. IADLs, instrumental activities of daily living. [Color figure can be viewed at wileyonlinelibrary.com]

Table 5 shows the additive interaction of IADLs and dual sensory on cognition based on logistic regression analysis. The risk of cognitive decline was 13.737 (95% CI: 9.726–19.400, *p* < 0.001) times higher in seniors with dual sensory difficulty and IADLs decline than in those with independent IADLs and no visual and hearing difficulty. The RERI was 7.823 (95% CI: 3.230–12.417), the AP was 0.570 (95% CI: 0.392–0.747), and S was 2.593 (95% CI: 1.616–4.160). According to Rothman,^22^ when the 95% CI of RERI and AP contained 0 and the 95% CI of S contained 1, there was no additive interaction; therefore, in this study, dual sensory difficulty and IADLs decline had an additive effect on cognition. Figure 3 shows the cognitive decline ORs, annotating the contributions of different exposure categories (IADLs decline and dual sensory difficulty).

**Table 5:** Additive interaction of IADLs and dual sensory on cognition.

IADLs decline	Dual sensory difficulty	*B*	*p*	OR (95% CI)
Yes	No	1.484	<0.001***	4.412 (3.633–5.358)
No	Yes	0.917	0.008*	2.502 (1.272–4.921)
Yes	Yes	2.620	<0.001***	13.737 (9.726–19.400)

Abbreviations: CI, confidence interval; IADLs, instrumental activities of daily living.

* *p* < 0.05;

*** *p* < 0.001.

## 4 DISCUSSION

### 4.1 The incidence of cognitive decline in the elderly

In previous studies, the incidence of cognitive decline in the elderly ranged from 6.7% to 29.94%.^24^, ^25^, ^26^, ^27^ In this study, the incidence of cognitive decline in the elderly was 26.32%. The differences in incidence rates between different studies may be related to the differences in the distribution of basic characteristics of the study population, and also the selection of different instruments to measure variables.

### 4.2 Risk factors of cognitive decline in the elderly

Due to the differences in literacy and socioeconomic background between women and men, women are more likely to suffer depression, anxiety, and other mental health problems, which might affect their cognitive function.^28^, ^29^ In addition, the decrease in estrogen in women after menopause also leads to cognitive function decline;^30^ therefore, women are more likely to develop cognitive decline, and this finding is consistent with the study results of Miyawaki and Liu.^28^ Ramos et al.^31^ showed that lower levels of literacy were associated with cognitive decline; the reason might be a lack of knowledge and skills, which negatively affects their cognitive function. In this study, participants who denied exercise habits were associated with cognitive decline, which is consistent with previous studies.^1^, ^32^, ^33^

Compared with the elderly who reported good health status, the elderly who reported poor health status were more likely to suffer from cognitive decline, which might be related to the higher frequency of social activity participation in the elderly with self‐reported good health. A higher frequency of social activity participation can promote the physical and mental health of the elderly, as well as the health of cognitive function.^34^, ^35^

### 4.3 Interaction effect of IADLs and dual sensory function on cognition

IADLs are advanced abilities that individuals need to live independently. A decline in IADLs means that individuals have difficulty performing complex tasks such as problem‐solving, multitasking, and decision‐making. The results of this study indicated that the elderly with IADLs decline were 4.635 times more likely to have cognitive impairment than those with independent IADLs. This finding was consistent with the results of previous studies,^11^, ^36^ which suggest that the decline of IADLs may be an indicator of potential cognitive impairment. Several studies^13^, ^14^, ^15^ have confirmed that visual and hearing difficulties in seniors increase the risk of cognitive impairment, possibly because visual and hearing difficulties limit the ability of seniors to receive and process information and communicate; it is also because those with visual and hearing difficulties have difficulty remembering or recalling information as easily as normal individuals, and were therefore at increased risk for cognitive impairment.

The results of the interaction in this study suggest that there is no multiplicative interaction between IADLs and dual sensory function on cognitive decline in seniors. Rothman^22^ has suggested that biological interaction should be analyzed using an addictive model rather than the multiplicative model. Results of this study examined that there was a strong additive interaction between the effects of IADLs decline and dual sensory difficulty on cognitive decline, with 57.00% of the risk of cognitive decline in seniors being attributed to an additive interaction between IADLs decline and dual sensory difficulty. Therefore, early screening of seniors for IADLs and sensory function is necessary, and the results of the screening can prompt early intervention in high‐risk populations and may reduce the risk of cognitive impairment significantly.

## 5 CONCLUSION

This study examined that IADLs decline and dual sensory difficulty are associated with cognitive decline in the elderly, and the result of interaction analysis explained that the coexistence of IADLs decline and dual sensory difficulty has a greater effect on cognitive decline in seniors. The results suggest that sensory and IADLs assessment should be used as early cognition screening items; through the sensory and IADLs screening, the cognition of high‐risk groups can be further assessed and can improve their awareness of active treatment. In addition, the protection of sensory and IADLs in the elderly are of great significance for the protection of their cognitive ability. In conclusion, the results of this study provide new ideas and research directions for protecting the cognition of the elderly. Samples of this study were limited to Zunyi City, Guizhou Province, China. In the future, a multicenter large‐sample survey can be conducted nationwide to improve the generalizability of the results.

## AUTHOR CONTRIBUTIONS

Heting Liang and Xiaoli Yuan contributed to the main conception and data collection, polished the manuscript, and analyzed most of the data. Xiaoli Yuan is responsible for quality control and is responsible for the whole article. Zhixia Jiang has confirmed the feasibility of the study and has read and approved the final submitted manuscript. Xiaoling Yang, Shuang Li, Xiaoling Zhao, Yongya Dai, Siqin Liu, and Yumeng Zhang were responsible for data collation, interpretation, and verification of statistical analysis results.

## CONFLICT OF INTEREST STATEMENT

The authors declare no conflict of interest.

## ETHICS STATEMENT

This study was approved by the Ethics Committee of the Affiliated Hospital of Zunyi Medical University on October 28, 2022 (No. KLL2022‐814).

## Data Availability

Data within this article is available and can be obtained on request.
